# Alfaxalone is an effective anesthetic for the electrophysiological study of anoxia-tolerance mechanisms in western painted turtle pyramidal neurons

**DOI:** 10.1371/journal.pone.0298065

**Published:** 2024-04-16

**Authors:** Haushe Suganthan, Domenic Di Stefano, Leslie T. Buck

**Affiliations:** 1 Department of Cell and Systems Biology, University of Toronto, Toronto, Ontario, Canada; 2 Department of Ecology and Evolutionary Biology, University of Toronto, Toronto, Ontario, Canada; Doheny Eye Institute/UCLA, UNITED STATES

## Abstract

Anoxia in the mammalian brain leads to hyper-excitability and cell death; however, this cascade of events does not occur in the anoxia-tolerant brain of the western painted turtle, *Chrysemys picta belli*. The painted turtle has become an important anoxia-tolerant model to study brain, heart, and liver function in the absence of oxygen, but being anoxia-tolerant likely means that decapitation alone is not a suitable method of euthanasia. Many anesthetics have long-term effects on ion channels and are not appropriate for same day experimentation. Using whole-cell electrophysiological techniques, we examine the effects of the anesthetic, Alfaxalone, on pyramidal cell action potential amplitude, threshold, rise and decay time, width, frequency, whole cell conductance, and evoked GABA_A_ receptors currents to determine if any of these characteristics are altered with the use of Alfaxalone for animal sedation. We find that Alfaxalone has no long-term impact on action potential parameters or whole-cell conductance. When acutely applied to naïve tissue, Alfaxalone did lengthen GABA_A_ receptor current decay rates by 1.5-fold. Following whole-animal sedation with Alfaxalone, evoked whole cell GABA_A_ receptor current decay rates displayed an increasing trend with 1 and 2 hours after brain sheet preparation, but showed no significant change after a 3-hour washout period. Therefore, we conclude that Alfaxalone is a suitable anesthetic for same day use in electrophysiological studies in western painted turtle brain tissue.

## Introduction

The western painted turtle, *Chrysemys picta bellii*, is unique in withstanding prolonged periods of anoxia, with no known loss of physiological function following 4 months in severely hypoxic conditions at 3°C [1–3]. The ability to survive severe hypoxia for extensive periods of time has made it a model species for hypoxia tolerance researchers. Alternately, the mammalian brain is extremely sensitive to even mild hypoxia, a time in which cellular ATP cannot be maintained, leading to Na^+^/K^+^ ATPase pump failure, and neuronal membrane potential becomes unstable and decreases dramatically. This results in anoxic depolarization, leading to electrical hyper-excitability through deranged glutamatergic signalling and excessive, deleterious Ca^2+^ influx through the chronic activation of N-methyl-D-aspartate (NMDA) receptors, resulting in neuronal excitotoxic cell death (ECD) [4]. Understanding the anoxia tolerance mechanisms in anoxia-tolerant species has translational value in suggesting strategies to reduce low-oxygen-related tissue injury in humans, particularly following cardiac infarctions (heart attacks), strokes, and cardiopulmonary disorders [1, 2, 4].

When harvesting tissues from animals to perform experiments, it is important to consider an appropriate anesthetic to render the animal unconscious. Many anesthetics work by depressing cell excitability through inhibiting or activating ion channels; hence, when studying neurons and their function, the anesthetic can act as a confound to what one is examining [5]. In addition to this, anesthetics used to euthanize animals for in-vitro electrophysiology experiments can have a relatively long washout time. In a study conducted by Keifer and Zheng (2017) the anesthetic—tricaine methanesulfonate (MS222) was delivered to turtles using intraperitoneal injection, and once anesthetized their brain stems were surgically isolated. After a 3–5-hour *in-vitro* washout, electrical stimulation of the trigeminal nerve to evoke the neural correlate of an eyeblink reflex, depicted very little spiking activity. The next day, recordings were consistent with the day before, showing very weak to no evoked reflex response. Another study investigated the extraocular motor discharge of xenopus tadpoles anesthetized with MS222, via spontaneous and evoked mechanosensory nerve activity, these isolated semi-intact *in-vitro* preparations required a 24-hour washout prior to recording any neural activity, which suggested a strong depression of brain activity after MS222 treatment [6].

Most anesthetic agents modulate ion channels, such as NMDA or Gamma-aminobutyric acid (GABA) receptors; thus, when looking at neuroprotective effects associated with these channels, it can interfere with the outcome of experiments [5]. Examples include isoflurane, where its anesthetic effect is mediated by antagonizing NMDA receptors and by potentiating GABA_A_ receptors. Ketamine is known to produce anesthesia by acting as an NMDA receptor antagonist through blockage of the channel pore; therefore, blocking excitatory synaptic activity, and at high doses ‐ blocking sodium channels. Barbiturates mediate their anesthetic action by acting as agonists of GABA_A_ receptors [5]. Other important factors to account for when considering the appropriate anesthetic is their metabolism, retention, and clearance times. Most anesthetics have long-lasting effects and can interfere with many aspects of cellular mechanisms associated with hypoxia/anoxia tolerance [7]. A review looking into anesthetics in reptiles and general recovery time alludes to most anesthetic agents requiring many hours to days to metabolize and clear from tissues [8–11]. Mammalian metabolic rate is about ten times higher than that of ectothermic vertebrates, such as turtles; therefore, when analyzing anesthetic metabolism, the reduced metabolic rate in reptiles must be taken into account [12, 13]. One drug that requires relatively smaller dosages and has a significantly faster recovery/clearance time in mammals and reptiles is Alfaxalone [8, 14].

Decapitation has been widely used as a method to euthanize turtles, in which the brain is subsequently removed, and cerebral cortical sheets are prepared for electrophysiological experiments. Although it’s a relatively quick procedure, the turtle’s extreme anoxia tolerance has generated concern regarding the pain that the animal may experience during and after decapitation. Therefore, the fast onset and washout of Alfaxalone makes it a potentially useful anesthetic agent for western painted turtle euthanasia. Alfaxalone was introduced as an anesthetic in the 1970’s and was used in conjunction with a weaker anesthetic ‐ Alfadolone [15]. It was removed from the market soon after its release due to the adverse effects it had on both felines and canines, producing allergic reactions [15]. In 2011, Canada approved a new formulation of Alfaxalone that has proved to be safe and effective to use as an anesthetic. Alfaxalone is a synthetic, neuroactive steroid that binds to an allosteric site on the GABA_A_ receptor, reducing its closing time and increasing Cl^-^ conductance, which can lead to shunting inhibition [16]. Whole-cell patch-clamp recordings from cultured rat hippocampal neurons also showed Alfaxalone potentiated the Cl^-^ conductance of GABA_A_ receptor currents [17] and increased decay time of spontaneous, inhibitory post-synaptic currents [18].

Alfaxalone use in both mammals and reptiles is known to have a smooth induction and rapid recovery [19, 20]. It is an injectable anesthetic, as opposed to commonly used inhaled anesthetics, which are difficult to use on turtles due to their strong head and neck withdrawal, and the ability of turtles to hold their breath for extensive periods of time [21]. Knotek et al. (2015) conducted a study with 77 chelonians (turtles): 33 red-eared terrapins (*Trachemys scripta elegans*), 11 Hermann´s tortoises (*Testudo hermanni*), 8 spur-thigh tortoises (*Testudo graeca*), 10 marginated tortoises (*Testudo marginata*), 12 Russian tortoises (*Testudo horsfieldi*), and 3 African spur thigh tortoises (*Geochelone sulcata*), which were all given intravenous Alfaxalone (Alfaxan® 10 mg/ml) at a dose of 5 mg/kg. Alfaxalone performed well as an induction agent in this study with a rapid onset (35–40 second loss of deep pain sensation), and a rapid, full recovery approximately 30 minutes post-induction with full restoration of activity. Another study looking at pharmacodynamics of Alfaxalone in red-eared slider turtles showed the longest recovery time to be 257 minutes for those administered with 20 mg/kg within an 18–20°C temperature range, and the shortest to be 126 minutes within 22–25°C for those administered with 10 mg/kg. Whereas cats receiving 5 mg/kg had a recovery time of about 45 minutes, and dogs receiving 6 mg/kg had a recovery time of about 30 minutes, likely resulting from a more rapid metabolism in mammals [19, 20]. In other reptiles, such as the ball python and green iguana intramuscular administration of three different doses of Alfaxalone resulted in a maximum effect within 10 minutes [22, 23] James et al., 2017. Similarly, Knotek et al. (2013) looked at short-term anesthesia with Alfaxalone in green iguanas at a dose rate of 5 mg/kg, and found both rapid induction, where loss of the toe-pinch reflex occurred at 2.3 minutes, and rapid recovery to full activity in about 14 min after injection.

Alfaxalone is a fast onset and offset anesthetic that may be promising for use in anoxia-tolerant species, such as the western painted turtle, where decapitation alone, is not sufficient for tissue harvesting. Therefore, the aim of this study is to determine if Alfaxalone sedation has any long-lasting effects on action potential properties or GABA_A_ receptor currents, as well as to investigate whether it is suitable for use on the same day as the electrophysiological studies are conducted on the painted turtle.

## Methods and materials

### Animal care and cortical sheet preparation

This study was approved by the University of Toronto Animal Care Committee and conforms to the care and handling of animals as outlined in the Canadian Council on Animal Care’s Guide to the Care and Use of Experimental Animals, Vol. 2. Animal use protocol number (20012745). Informed Consent Statement: Not applicable. The protocol described in this peer-reviewed article is published on protocols.io doi.org/10.17504/protocols.io.x54v9pzjzg3e/v1 and is included for printing purposes as S1 File. Adult turtles (*Chrysemys picta bellii*) were obtained from Niles Biological and housed in a large aquarium equipped with a freshwater recirculating filter system set at 20°C, a non-aquatic basking platform, and a heat lamp. Turtles were maintained on a photoperiod of 12 hours of light:12 hours of dark and given access to food three times a week. Turtles were given an intramuscular injection of Alfaxalone 10 mg/mL stock in hydroxypropyl-beta-cyclodextrin [HP-β-CD] and dissolved in a multi-dose preservative solution (Alfaxan®) (Jurox Pty Ltd; Rutherford, Australia) at 20 mg/kg. Once deep anesthesia was achieved, the legs were flaccid, and the eyes were unresponsive to gentle touch, the animals were then decapitated with a guillotine, and the whole brain was excised from the cranium in about 1 minute. Naïve brain tissue was gathered from turtles that were not exposed to Alfaxalone prior to decapitation. The entire dorsal cortex was dissected free and bathed in 3–5°C artificial turtle cerebrospinal fluid (aCSF) composed of (in mmol L –^1^): 97 NaCl, 2.6 KCl, 1.2 CaCl_2_, 1.0 MgCl_2_, 2.0 NaH_2_PO_4_, 26.5 NaHCO_3_, 20.0 glucose, 5.0 imidazole (adjusted to pH 7.4, and osmolarity 290–300 mOsM). Two cortical sheets were cut medially from the visual cortex of each cerebral hemisphere and subdivided into a total of six cortical sheets. Sheets were then lifted out of the chamber and stored in vials of aCSF for no longer than 48 h [24].

### Whole-cell electrophysiology techniques

Turtle cortical sheets were placed on a cover slip that forms the bottom of an RC-26 open bath perfusion chamber system, with a P1 platform (Harvard Apparatus, Saint-Laurent, QC, Canada). The chamber was gravity-perfused by a 1-L glass bottle that contained aCSF gassed with 95% O_2_/5% CO_2_ to achieve oxidative conditions. Experiments were conducted at room temperature (20–22°C). Whole-cell recordings of neurons from the dorsal cortex and dorsal medial cortex were performed using fire-polished 5–8 MΩ micropipettes produced from borosilicate glass capillary tubes using a P-97 micropipette puller model (Sutter Instruments, Novato, CA, USA). The pipette solution contained the following (in mmol L^-1^): 8 NaCl, 0.0001 CaCl_2_, 10 Na-HEPES, 110 Kgluconate, 1 MgCl_2_, 0.3 NaGTP, and 2 NaATP (adjusted to pH 7.4 and osmolarity 290–300 mOsM). The electrode was filled and inserted into a 1-HL-U electrode holder attached to a CV-4 headstage (gain: 1/100 U, Axon Instruments, Sunnyvale, CA, USA)

Cell-attached 1–20 GΩ seals were obtained using the blind-patch technique [25]. To achieve a GΩ seal, the recording electrode was advanced towards the cell using a PCS-6000 motorized manipulator (Burleigh, Newton, NJ, USA) until the square-wave pulse abruptly decreased, at which point a slight negative pressure was applied to form a seal. To break into the cell, a soft pulse of negative pressure was applied to break through the cell membrane, while the holding potential was voltage-clamped to -70 mV. Once the whole-cell configuration was established, cells were given at least 2 minutes to acclimate to experimental conditions before access resistance was measured, which normally ranged from 20–30 MΩ. Patches were discarded if access resistance varied by >25% over the course of an experiment. Data was collected at 5–10 kHz using a MultiClamp 700B digital amplifier, a CV-7B head stage, and a Digidata 1550B interface (Molecular Devices, Sunnyvale, CA, USA), and stored on a computer using Clampex 10 software (Molecular Devices, Sunnyvale, CA, USA). A liquid junction potential (LJP) was accounted for, and experimentally measured between the aCSF and the pipette solution, supported by LJP calculations using a generalized version of the Henderson equation (Clampex junction potential calculator; Molecular Devices, Sunnyvale, CA, USA) [24, 26].

### Electrophysiological identification and measurement of action potential parameters

**Fig 1:**
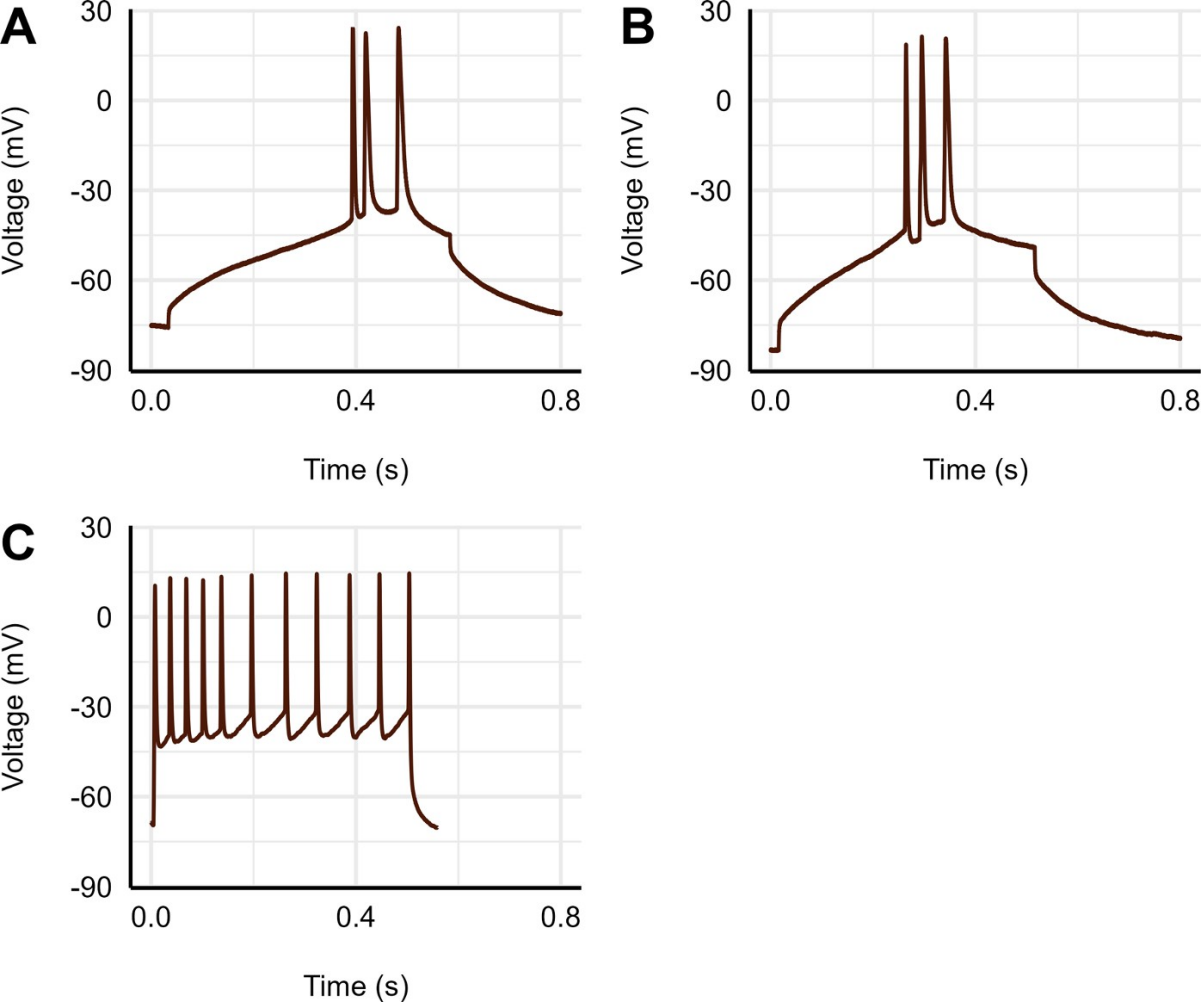
Evoked action potential recordings. AP traces generated from 500 ms current steps in 10 pA increments from naïve tissue **(A)** naïve pyramidal neuron, **(B)** a pyramidal neuron from a painted turtle tissue following whole-animal Alfaxalone sedation, and **(C)** stellate neuron.

Pyramidal neurons were studied and characterized based on electrophysiological properties. In current clamp mode, when current was injected, pyramidal neurons exhibited spike frequency adaptation in response to a sustained current, which was not seen in stellate neurons (Fig 1) [27].

Action potential threshold (APth) was determined by current-clamping cells and injecting current in 10 pA increments in a stepwise manner from sub-threshold for 500 ms until a spike was elicited. The threshold was recorded at the point at which a sharp elevation in voltage was observed. The full spike amplitude was measured from the point of the APth to the spike tip, while the half-amplitude spike width was measured as the time elapsed between the two-points of the half-amplitude on the spike. The rise time was calculated as the time elapsed between 10% of the full spike amplitude and 90% of the full spike amplitude, while the decay time was calculated as the time elapsed between 10% of the full spike amplitude and 90% of the full spike amplitude. Whole-cell conductance (Gw) was measured as the slope of a voltage-ramp from -120 to -60 mV for 150 ms. Data measurements for all parameters were made with Clampfit software (Molecular Devices, Sunnyvale, CA, USA) [24].

#### Impact of acute Alfaxalone application on evoked naïve tissue GABA_A_ Receptor current

To initiate a GABA_A_ receptor current neurons were voltage-clamped at a holding potential of -100 mV, and 2 mM of GABA were applied for 1–2 seconds [26]. These changes resulted in large outward GABA_A_- receptor currents that were easily detected and differentiated from other currents. From the GABA_A_ receptor currents produced, we measured the peak amplitude, baseline holding current, decay time as the 90%-to-10% decay time, and the area under the curve as the integrated area between the measured current and the baseline using Clampfit software (Molecular Devices, Sunnyvale, CA, USA). Sheets were then perfused with oxygenated aCSF and 1 μM Alfaxalone for 15 min, and the GABA_A_ receptor current decay time, integrated area under the curve, peak amplitude, and baseline holding current were measured again. This procedure was repeated utilizing gabazine (25 μM), a GABA_A_ receptor antagonist, rather than Alfaxalone, to see if the currents could be blocked to confirm we were recording GABA_A_ receptor currents [26]. Additionally, Alfaxalone-free stock Alfaxalone solution (vehicle, a generous gift from Jurox; Rutherford, NSW, Australia) had no significant impact when applied alone (S1 Fig and S1 Dataset) (n = 4).

To construct a dose-response curve, GABA_A_ receptor current measurements following acute Alfaxalone treatment were normalized to pre-treatment values to determine the relative change in decay time, area under the curve, and peak amplitude. The same process to determine relative changes in decay time, area under the curve, and peak amplitude was repeated for tissue sheets perfused with oxygenated aCSF and 0.1 μM, 0.5 μM, 1 μM or 1.5 μM Alfaxalone for 15 min (S2 Fig and S1 Dataset). Data was fit to the 4-parameter Hill equation (log-logistic equation) to construct a dose response curve for each of the parameters [28].

#### Impact of whole-animal Alfaxalone exposure on evoked GABA_A_ receptor whole cell current

GABA_A_ receptor currents were measured as above; however, the recording pipette [Cl^-^] was increased to 110 mM [Cl^-^] by equimolar substitution of KCl for Kgluconate, the neurons were voltage-clamped at a holding potential of -100 mV and 2 mM GABA was applied for 15 seconds [26]. This change still resulted in large outward GABA_A_ currents that were detected and differentiated from other currents. The GABA_A_ receptor current decay time, integrated area under the curve, and peak amplitude were determined as described above. The decay time, integrated area under the curve, peak amplitude, and baseline holding current were then normalized to the whole-cell capacitance. The protocol was repeated with cerebral cortex sheets obtained from Alfaxalone-sedated painted turtles at 1 hour, 2 hours, 3 hours, and 5 hours following Alfaxalone administration. The timepoints were binned so that any measurement performed between 1 and 2 hours was considered 1 hour following whole-animal Alfaxalone exposure, any measurement performed between 2 and 3 hours following whole-animal Alfaxalone exposure was considered 2 hours following whole-animal Alfaxalone exposure, any measurement performed between 3 and 4 hours was considered 3 hours following whole-animal Alfaxalone exposure, and any measurement performed between 5 and 6 hours was considered 5 hours following whole-animal Alfaxalone exposure. Measurements made on the 30-minute mark were placed in the latter time group. The aCSF solution bathing the Alfaxalone-treated sheets were replaced every 30 minutes following the completion of the dissection with the first washout occurring 0.5 hours after Alfaxalone application.

### Statistics

Welch’s t-tests were conducted to compare AP electrophysiological property values between treatments. AP traces that did not immediately return to baseline or hyperpolarize were excluded, as decay time could not be determined accurately. Paired t-tests were conducted to compare normalized GABA_A_ receptor active and passive electrophysiological property values between treatment groups. Welch’s t-tests were also conducted to compare GABA_A_ receptor electrophysiological property values at various time points to control values. N values represent a recording from a single cell from a single cerebral cortex sheet from a single painted turtle. P < 0.05 was considered statistically significant. All statistical analyses were performed using R.

## Results

### Differentiation of pyramidal and stellate neurons

To generate action potentials (APs) 500 ms current steps in 10 pA increments were injected into cells, and voltage traces were obtained until APs were generated, at which point current injection was halted. Voltage traces containing one or multiple APs were then isolated for analysis. Current clamp recordings were conducted in control (no Alfaxalone exposure) and whole-animal Alfaxalone-treated tissues, as shown in Fig 1, respectively. Pyramidal cell voltage traces were characterized by low frequency APs that lacked accommodation. Stellate neuron traces fired APs during the entire current injection phase (Fig 1) and were not included in the present analysis.

### The effects of Alfaxalone on AP properties

**Fig 2:**
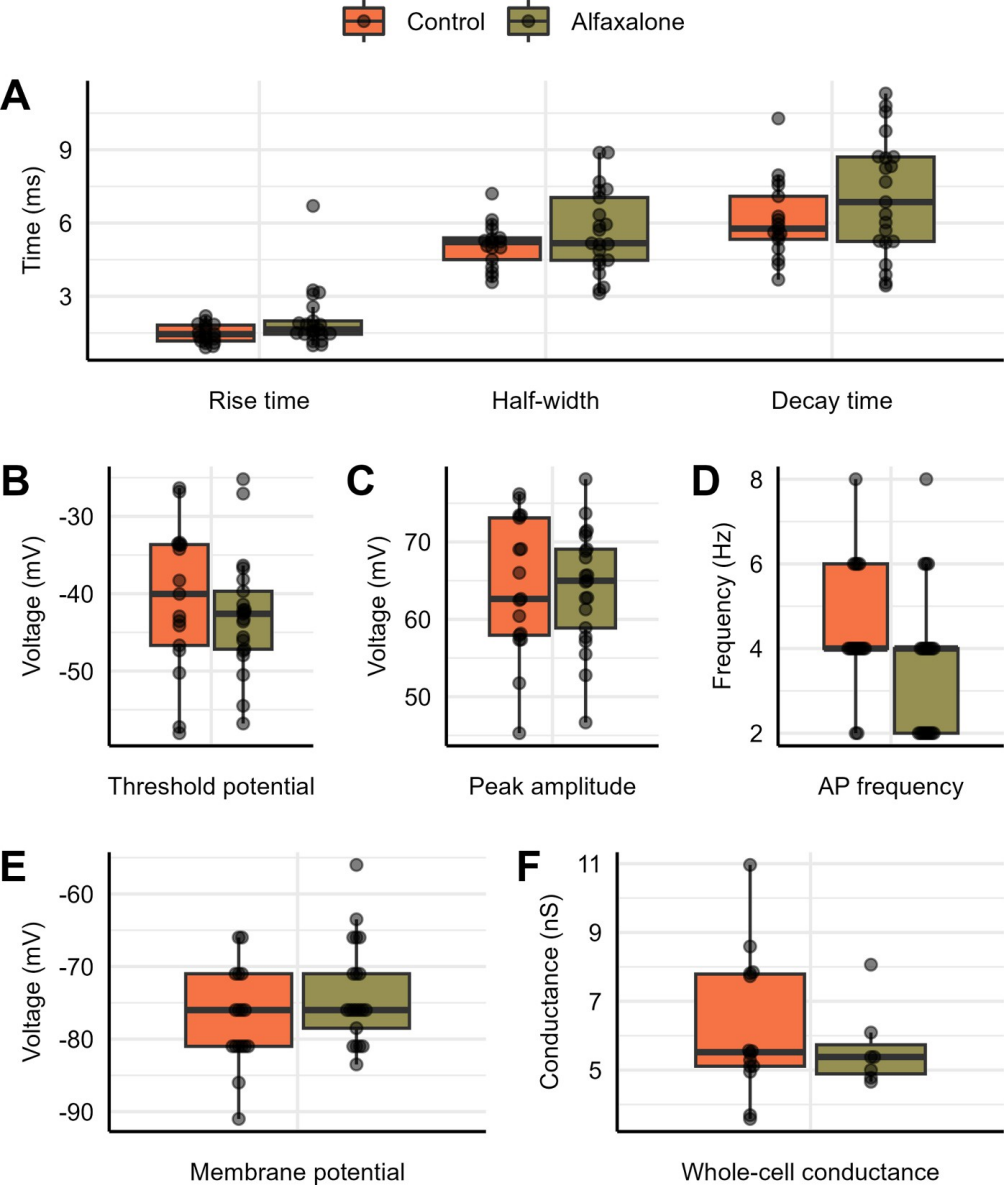
Action potential properties and whole cell conductance for control and whole-animal Alfaxalone-sedated painted turtle brain tissue. Properties include: **(A)** action potential half-width (ms), rise time (ms), and decay time (ms) (*n* = 17 and 21); **(B)** threshold potential (mV) (*n* = 17 and 21); **(C)** peak amplitude (mV) (*n* = 17 and 21); **(D)** action potential frequency (Hz) (*n* = 17 and 21); **(E)** baseline potential (mV) (*n* = 17 and 21); and **(F)** whole-cell conductance (ns) when ramping the cell from -120 mv to -60 mv (*n* = 17 and 21) between the control and Alfaxalone-sedated western painted turtle cortical pyramidal neurons. Each point represents data from a separate experiment and n values are reported in terms of control and Alfaxalone-treated tissue, respectively. Statistical significance was determined using Welch’s t-test.

To obtain AP electrophysiological characteristics from control and Alfaxalone-treated tissue, data was analyzed using the program Clampfit. In turtle pyramidal neurons the threshold for the control was -40.6 ± 9.42 mV, and -42.7 ± 7.57 mV for the Alfaxalone-treated (n = 17, 21, respectively; P = 0.411) (Fig 2). The membrane potential for the control was -77.18 ± 6.74, and for the Alfaxalone-treated it was -73.74 ± 7.02 (n = 17, 21, respectively; P = 0.191) (Fig 2). Action potential frequency, which refers to the number of spikes per defined time, was 4.47 ± 1.5 Hz for control, and 3.62 ± 1.75 Hz for the Alfaxalone-treated (n = 17, 21, respectively; P = 0.085) (Fig 2). Peak amplitude for the control was 64.08 ± 8.93 mV and 64.18 ± 7.55 mV for the Alfaxalone-treated (n = 17, 21; P = 0.490) (Fig 2). Half-width under control conditions was 5.09 ± 0.91 ms and 5.63 ± 1.73 ms for the Alfaxalone-treated (n = 17, 21, respectively; P = 0.499) (Fig 2). Rise time for the control was 1.47 ± 0.38 ms and 2.05 ± 1.26 ms for the Alfaxalone-treated (n = 17, 21, respectively; P = 0.113) (Fig 2). Decay time for the control was 6.13 ± 1.61 ms and 7.07 ± 2.45 ms for the Alfaxalone-treated (n = 17, 21, respectively; P = 0.265) (Fig 2). No statistical differences were found between the control and Alfaxalone-treated tissue for any of the electrophysiological properties, as demonstrated through Fig 2 and Table 1 (S1 Dataset).

**Table 1:** Control and Alfaxalone treated tissue AP electrophysiological property summary values. Data are mean ± s.e.m.

Property	Control	Alfaxalone	P-value	Historical Values (Hawrysh and Buck., 2019)
**Rise time (ms)**	1.47 ± 0.38 (17)	2.05 ± 1.26 (21)	0.113	
**Half-width (ms)**	5.09 ± 0.91 (17)	5.63 ± 1.73 (21)	0.499	3.0 ± 0.2 (24)
**Decay time (ms)**	6.13 ± 1.61 (17)	7.07 ± 2.45 (21)	0.265	
**Threshold potential (mV)**	-40.6 ± 9.42 (17)	-42.7 ± 7.57 (21)	0.411	−37.7 ± 2.1 (15)
**Peak amplitude (mV)**	64.08 ± 8.93 (17)	64.18 ± 7.55 (21)	0.490	58.5 ± 2.7 (24)
**Action potential frequency (Hz)**	4.47 ± 1.5 (17)	3.62 ± 1.75 (21)	0.085	
**Membrane potential (mV)**	-77.18 ± 6.74 (17)	-73.4 ± 7.02 (21)	0.191	−88.4 ± 0.9 mV (4)
**Whole cell conductance (pS)**	6.67 ± 0.55 (12)	5.67 ± 0.37 (10)	0.526	4.8 ± 0.3 (34)

To determine whole-cell conductance values for the control and Alfaxalone-treated tissue, voltage clamp recordings were conducted using a voltage ramp protocol, increasing the voltage from -120 mV to -60 mv, while measuring the corresponding current. Voltage and current measurements were plotted against each other in a current-voltage (I-V) curve. Linear regression of the I-V curves was used to generate a line of best fit, and the slope was used to determine whole-cell conductance values. Whole cell conductance for the control neurons was 6.67 ± 0.55 pS and 5.67 ± 0.37 pS for the Alfaxalone-treated (n = 12, 10, respectively; P = 0.526) (Fig 2). No statistical differences were found between the control and Alfaxalone-treated tissue whole-cell conductance values.

### Evoked GABA_A_ receptor currents

**Fig 3:**
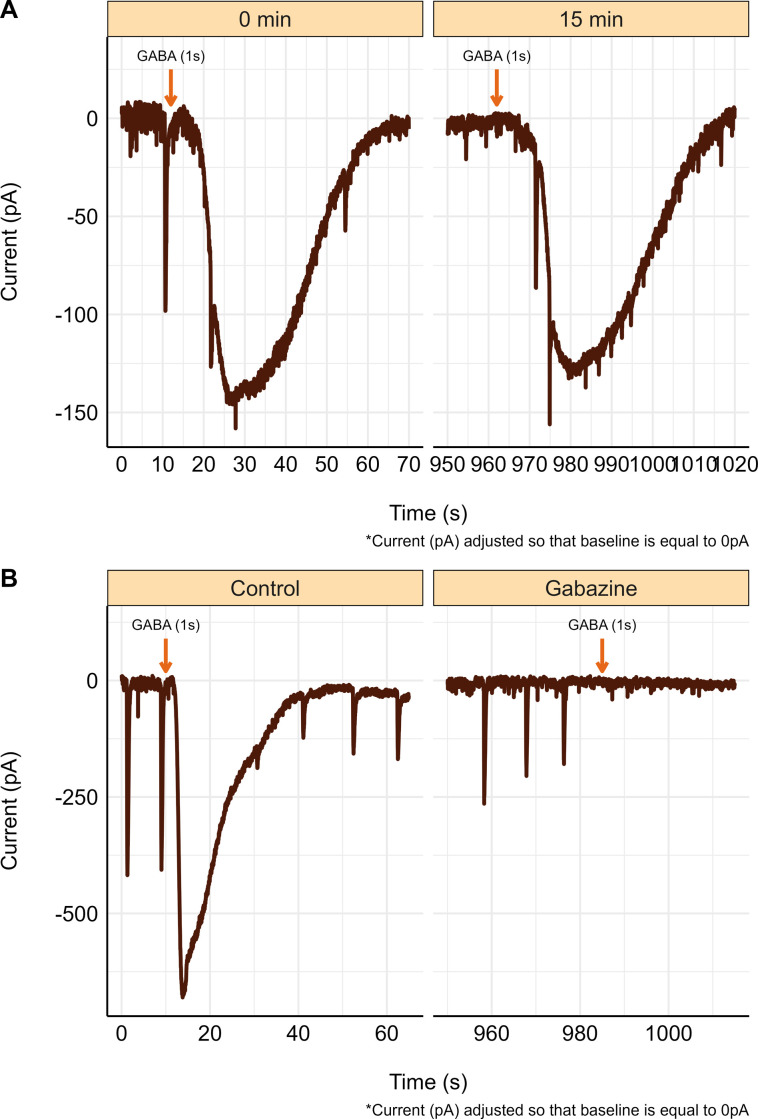
Whole-Cell GABA_A_ receptor currents and stability in neurons from naïve tissue. **(A)** Whole-cell GABA_A_ receptor currents, following GABA (2 mM) application, taken 15 minutes apart with no change in evoked current characteristics. **(B)** GABA_A_ receptor current trace following GABA (2 mM) application under control conditions and following 15 minutes of Gabazine (25μM) perfusion. Gabazine perfusion began immediately following the completion of the initial control GABA_A_ receptor current, then GABA was perfused for 1–2 seconds, where no current was evoked. Note the disappearance of spontaneous GABA_A_ currents in panel (B) after gabazine application.

To generate a whole-cell GABA_A_ receptor current trace, neurons were voltage-clamped at -100 mV, and then bath-perfused with 2 mM of GABA for 1–2 seconds. This process was repeated after 15 minutes using the same whole-cell patch to generate an additional GABA_A_ receptor current and confirm the stability of the whole-cell patch. Raw time 0- and 15-min GABA_A_ receptor currents are shown in Fig 3. To confirm that we were studying GABA_A_ receptor currents the antagonist gabazine (25 μM) was perfused into the recording chamber 15 minutes before GABA was applied. Following perfusion of Gabazine, GABA_A_ receptor currents could not be generated Fig 3. Note that spontaneous post-synaptic currents were also eliminated.

### Acute Alfaxalone application onto naïve tissue and impact on GABA_A_ receptor currents

**Fig 4:**
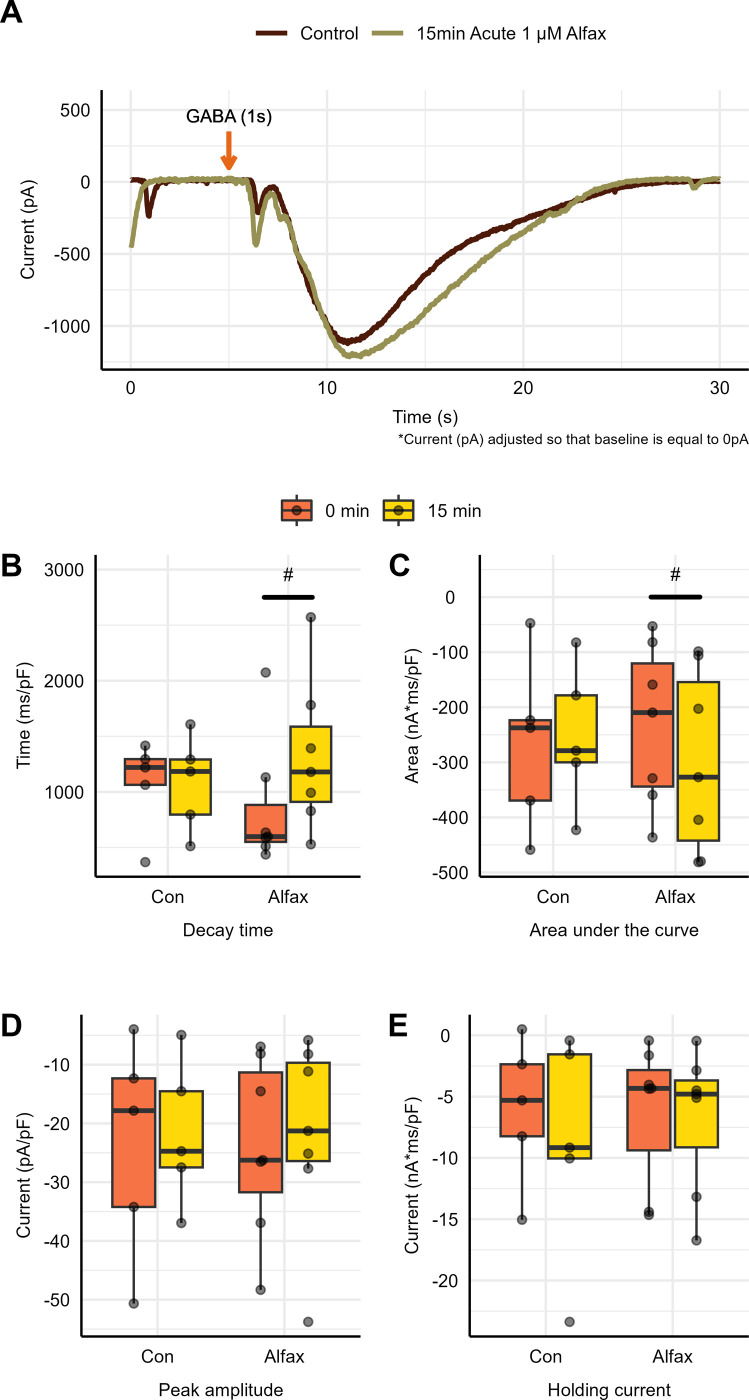
GABA_A_ receptor potentiation following acute application of Alfaxalone onto naïve tissue. **(A)** Change in whole cell GABA_A_ receptor response following Alfaxalone application onto naïve tissue. Change in **(B)** decay time, **(C)** area under the curve, **(D)** peak amplitude, and **(E)** holding current following 15 minutes of 1 μM Alfaxalone perfusion onto tissue. Each point represents data from a separate experiment (*n = 7*). Asterisks indicate significant difference from control conditions using a paired t-test (*P < 0.05). Note C and D show increases in negative current following Alfaxalone exposure.

To determine the impact of acute Alfaxalone exposure on GABA_A_ receptor currents, naive brain sheets were perfused with 1 μM Alfaxalone for 15 minutes and then GABA was applied to elicit a current (as above and Fig 4). After which current decay time, area under the curve, and peak amplitude under control conditions and following the acute application of Alfaxalone were determined. A statistically significant (P = 0.015) increase in decay time was found between the control (854 ± 220 ms/pF, n = 7) and acute Alfaxalone application (1320 ± 257 ms/pF, n = 7; Fig 4). A statistically significant (P = 0.025) difference in area under the curve was found between the control (-233 ± 55.1 nA·ms /pF, n = 7) and acute Alfaxalone application (-300 ± 62.6 nA·ms/pF, n = 7; Fig 4). No statistically significant (P = 0.347) difference in peak amplitude was found between the control (-23.8 ± 8.34 nA/pF, n = 7) and acute Alfaxalone application treatment groups (-21.7 ±5.51 nA/pF, n = 7; Fig 4 and S1 Dataset).

To determine if there was any impact of Alfaxalone on the holding current required to maintain a cellular membrane potential of -100 mV, current was measured under control conditions and following the acute application of 1 μM of Alfaxalone onto naïve tissue for 15 min. No statistically significant (-0.54 ± 0.39 pA/pF, n = 7, P = 0.224) difference in holding current was found between the control (-6.26 ± 2.21 nA/pF) and acute Alfaxalone application after 15 min (-6.80 ± 2.22 nA/pF; Fig 4). There was also no change in the holding current in naïve neurons held under control conditions for 15 min.

#### Alfaxalone washout time

To determine the washout period of Alfaxalone, GABA_A_ receptor currents were measured from control naïve tissue and tissue following whole-animal Alfaxalone sedation, as described in the Methods. At various time-points (1, 2, 3, and 5 hour(s)) following anesthetic administration and brain tissue dissection, current decay time, area under the curve, and peak amplitude were determined using the program ‐ Clampfit and normalized to whole-cell capacitance.

**Fig 5:**
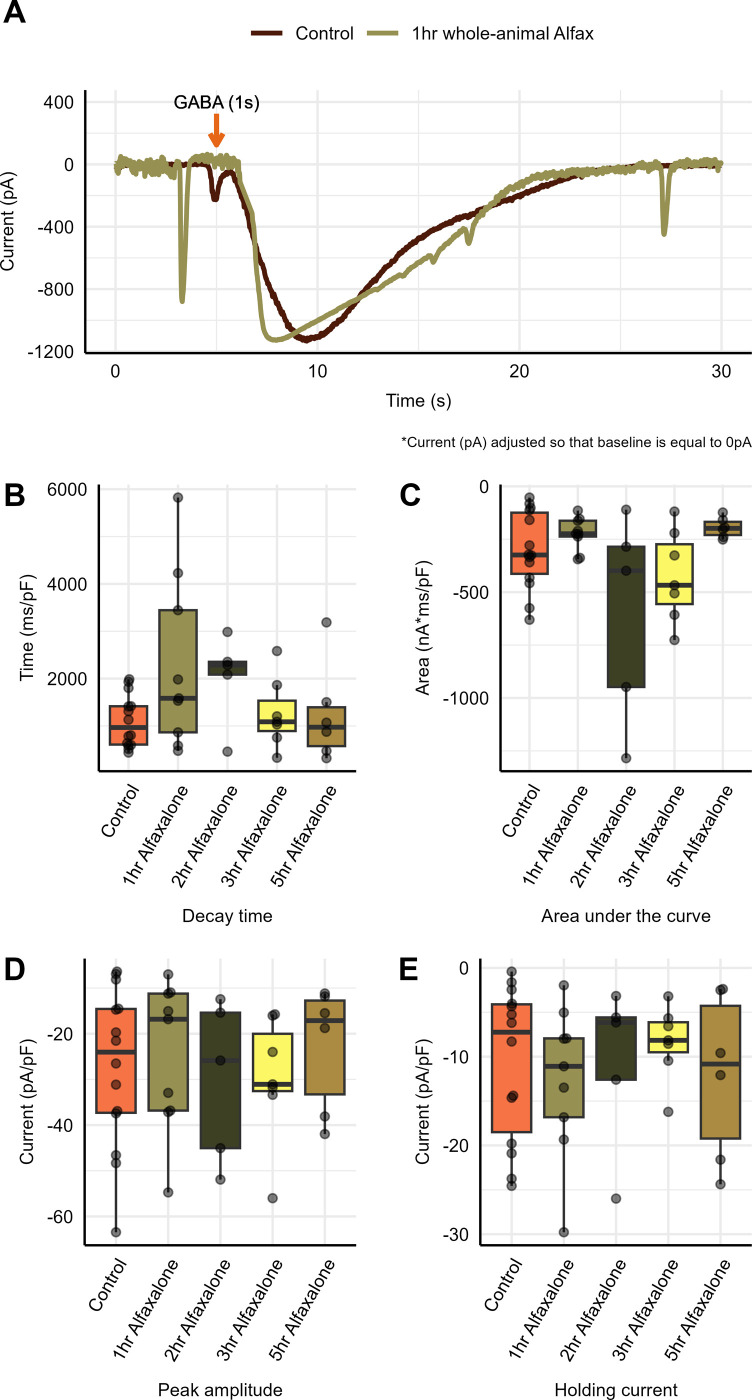
GABA_A_ receptor potentiation following whole-animal Alfaxalone sedation. GABA_A_ receptor current following GABA application under control conditions (no Alfaxalone sedation/exposure) and following 1, 2, 3, and 5 hours after whole animal Alfaxalone sedation. **(A)** GABA_A_ receptor current **(B)** decay time, **(C)** area under the curve, **(D)** peak amplitude and **(E)** holding current in control conditions (*n* = 14) and at 1 hour (*n* = 8), 2 hours, (*n = 5*), 3 hours (*n* = 7), and 5 hours (*n* = 6) following whole-animal Alfaxalone sedation. Each point represents data from a separate animal.

As shown in Fig 5, no statistically significant difference in decay time was found (P = 0.093) between the control (1100 ± 1ms/pF, n = 14) and 1 hour following whole-animal Alfaxalone sedation (1096 ± 146.7 ms/pF, n = 9), although there did appear to be an increasing trend. Furthermore, there were no statistically significant differences found at 2, 3 and 5 hours (2034 ± 421.7 ms/pF, n = 5, P = 0.089), (1264 ± 281.3 ms/pF, n = 7, P = 0.584) and (1238 ± 426.4 ms/pF, n = 6, P = 0.408), respectively (Fig 5 and S1 Dataset).

As shown in Fig 5, no statistically significant difference was accounted for (P = 0.093) in the area under the curve between the control (-301.7 ± 48.96 nA·ms/pF, n = 14) and 1 hour following Alfaxalone whole-animal sedation (-223.4 ± 25.96 nA·ms/pF, n = 9). This statistical insignificance in the area under the curve remained the same at 2, 3 and 5 hours following Alfaxalone sedation (303.7 ± 0.24 nA·ms/pF, n = 5, P = 0.243), (-424.6 ± 81.25 nA·ms/pF, n = 7, P = 0.389) and (-194.9 ± 19.66 nA·ms/pF, n = 6, P = 0.077), respectively (Fig 5).

As shown in Fig 5, no statistically significant difference was found (P = 0.724) in the peak amplitude between the control (-27.31 ± 4.67 nA/pF, n = 14) conditions and 1 hour following Alfaxalone sedation (-24.74 ± 5.42 nA/pF, n = 9). This statistical insignificance in peak amplitude remained the same at 2, 3 and 5 hours following Alfaxalone application (-30.13 ± 7.89 nA/pF, n = 5, P = 0.767), (-29.71 ± 5.18 nA/pF, n = 7, P = 0.732) and (-22.89 ± 5.56 nA/pF, n = 6, P = 0.386), respectively (Fig 5).

To determine whether Alfaxalone had any long-term impact on GABA_A_-R-mediated tonic currents, holding current was measured in control tissue and Alfaxalone-treated tissue at various time-points following anesthetic administration. Holding currents were measured and normalized to whole-cell capacitance. As shown in Fig 5, no statistically significant difference in holding current was found (P = 0.620) between the control (-10.76 ± 2.32 pA/pF, n = 14) conditions and 1 hour (-12.6 ± 2.83 nA/pF, n = 8) following Alfaxalone sedation. This statistical insignificance in holding current remained the same at 2, 3 and 5 hours following Alfaxalone application (-10.7 ± 4.13 nA/pF, n = 5, P = 0.9901), (-8.404±1.57 nA/pF, n = 7, P = 0.168), and (-12.09 ± 3.80 nA/pF, n = 6, P = 0.811), respectively (Fig 5).

## Discussion

Alfaxalone is a promising anesthetic agent for use in turtle brain and likely all reptile brain electrophysiological studies, as it is known to have rapid induction and recovery times in mammals [20]. We assessed pyramidal neuron action potential parameters, whole cell conductance, and GABA_A_ receptor whole-cell currents from brain sheets of both Alfaxalone-sedated turtles and turtles not sedated with Alfaxalone to validate its rapid onset and washout times. We found that the effects of whole-animal Alfaxalone sedation were reversed by 1 hour and that if naïve tissue was exposed to Alfaxalone in the recording chamber GABA_A_ receptor current decay time was increased. This positive control confirms that Alfaxalone does interact with the receptor in a predictable manner. Consequently, this also demonstrates that Alfaxalone is a rapid onset and offset anesthetic [16].

In this study, we investigated the impact of Alfaxalone sedation prior to decapitation on action potential electrophysiological characteristics using whole-cell patch recordings. We show that whole-animal Alfaxalone exposure has no impact on any of the evoked action potential properties, including rise time, decay time, half-width time, peak amplitude, threshold potential, alongside membrane potential, and whole cell conductance. Our results, therefore, support our hypothesis that intramuscular injection of Alfaxalone has no long-term impact on action potential parameters. The average membrane potential, whole-cell conductance, action potential threshold, and half-amplitude spike width values for the western painted turtle pyramidal neurons were previously reported to be -88.4 ± 0.9 mV and 4.8 ± 0.3 pS, -37.7 ± 2.1 mV, 3.0 ± 0.2 ms, respectively [24]. In comparison, our study found average membrane potential, whole-cell conductance, action potential threshold, and half-amplitude spike width values of -77.18 ± 6.74, 6.67 ± 0.55 pS, -40.6 ± 9.42 mV, and 5.09 ± 0.91 ms, respectively; and in control tissue 73.74 ± 7.02, 5.67 ± 0.37 pS, -42.7 ± 7.57 mV, and 5.63 ± 1.73 ms in Alfaxalone-sedated tissue, respectively (Table 1).

In addition, action potential rise and decay times in both Alfaxalone-sedated and control turtles were similar. Alfaxalone is a GABA_A_ receptor agonist and is not known to impact action potential properties that are determined by the action of voltage-sensitive fast sodium currents and outward potassium currents. Alfaxalone has not been found to alter Na^+^ and K^+^ channel structure or conductance following its application, and our results support this finding [29, 30].

We were surprised to find no residual effects of Alfaxalone on GABA_A_ receptor whole cell currents in tissue from animals sedated with it. There were no significant differences in peak current, rise time, or decay time 1-, 2-, 3-, or 5-hours following sedation, where the aCSF bathing the tissue was changed every 30 minutes. Although there was a trend in our data for decay time to appear to increase 1 and 2 hour(s) after Alfaxalone exposure, this trend was not statistically significant and is reversed by 3 hours.

We also determined the most effective concentration of Alfaxalone to be 1 μM, as shown in the dose-response relationship in S2 Fig. We identified that the increased decay time caused by acutely applied Alfaxalone becomes saturated at concentrations of approximately 1 μM. Effective whole-cell patch clamp recordings at an Alfaxalone concentration of 1 μM is within the range of concentrations 0.1–1 μM previously used in experiments with rat hippocampal neurons that increased in GABA_A_ receptor decay times, and area under the curve [17]. Analysis of decay time following acute application of Alfaxalone onto naïve tissue was limited at higher anesthetic concentrations. Concentrations of 2 μM and greater destabilized whole-cell patch recordings after approximately 10 minutes following acute Alfaxalone perfusion. Previous studies describe that Alfaxalone increases liposome membrane fluidity in correlation to anesthetic concentration [31]. The disturbance to membrane stability could potentially explain the destabilization at greater acute Alfaxalone treatment concentrations, thereby setting the upper limits of the dose-response curve.

As a positive control, we explored the short-term impact of Alfaxalone exposure on GABA_A_ receptor current characteristics by acutely perfusing Alfaxalone onto naïve tissue. Alfaxalone significantly increased GABA_A_ receptor current decay times and the area under the curve relative to control values; but there were no significant increases in peak amplitude and holding current (Fig 4). Our findings are consistent with previous studies where 1 μM Alfaxalone induced a short-term increase in GABA_A_ receptor decay times [32, 33]. Similar studies were done in rat hippocampal neurons with concentrations of Alfaxalone at 10 μM, which showed increased decay times and area for both spontaneous and evoked GABA-mediated inhibitory post-synaptic currents by 8–10-fold [17]. Harrison et al. (1987) further support our results, they showed similar results comparing decay time, peak amplitude, and area under the curve before and during Alfaxalone application, as decay times and area increased, whilst peak amplitude did not differ. Cao et al. (2018) similarly applied exogenous Alfaxalone in increasing concentrations, demonstrating significantly increased decay time in evoked-inhibitory post-synaptic currents (IPSCs) to enhance GABAergic effects. Therefore, these studies further support our results that Alfaxalone is a GABA agonist that prolongs decay time in IPSCs [17, 18]. In addition, Alfaxalone-free stock solution induced no significant impact when it was applied alone. Consistent with our results, the Alfaxalone solvent, 2-hydroxypropyl-β-cyclodextrin, has been shown to induce no significant impact when applied alone, following dilution by a factor of 1000 [34]. Stock Alfaxalone was diluted by a factor of at least 20000 in our study.

Overall, we conclude that Alfaxalone has no impact on action potential parameters, whole-cell conductance, or GABA_A_ receptor currents in the western painted turtle 3 hours following whole-animal sedation. Therefore, Alfaxalone is a promising anesthetic with fast induction and rapid washout for use in turtle brain analysis, and this conclusion is likely applicable to other reptiles and species.

## Conclusion

Alfaxalone has been identified as an anesthetic with a fast induction and quick washout in many animal models. In this study, our results demonstrate that intramuscular whole-animal Alfaxalone injection has no long-term impact on action potential properties or GABA_A_-receptor currents. Our results also confirm that Alfaxalone increases the decay time of GABA_A_ receptor currents following acute Alfaxalone perfusion onto naïve tissue. The use of an appropriate anesthetic to promote animal welfare is important, and Alfaxalone represents an anesthetic that is compatible with humane euthanasia and with same day dissection for electrophysiological studies in painted turtles and likely other reptiles.

## Supporting information

S1 File Step-by-step protocol, also available on protocols.io. (PDF)

S1 Fig GABA_A_ receptor current following acute application of Alfaxalone©-free stock Alfaxalone solution (Vehicle) onto tissue. Change in whole cell GABA_A_ receptor response following vehicle application onto tissue. Absolute change in **(A)** decay time, **(B)** area under the curve, **(C)** peak amplitude, and **(D)** holding current following 15 minutes of vehicle perfusion onto tissue. Each point represents data from a separate experiment (*n = 4*).
(TIFF)

S2 Fig Alfaxalone dose response curve. GABA_A_ receptor current measurements following acute Alfaxalone treatment normalized to pre-treatment values to determine the relative change in GABA_A_ receptor current **(A)** decay time, **(B)** area under the curve. **(C)** peak amplitude in naïve tissue sheets perfused with oxygenated aCSF and 0.1 μM (*n =* 3), 0.5 μM (*n =* 5), 1 μM (*n =* 7) and 1.5 μM (*n =* 3) Alfaxalone for 15 minutes, normalized to control GABA_A_ receptor decay time using the four-parameter Hill equation. The red point represents the average percent change in each property value following 15 minutes of control aCSF perfusion. Minimal peak amplitude potentiation occurred at increasing concentrations of Alfaxalone, resulting in a poor fit to the Hill equation. Alfaxalone concentrations greater than 1.5 μM resulted in patch destabilization within 10 minutes of perfusion. The Alfaxalone-induced potentiation of all properties saturated at approximately 1 μM with minimal patch destabilization. Therefore, it was determined that the acute application of 1 μM Alfaxalone was optimal and would be perfused onto naïve tissue for the remainder of the study. GABA_A_ receptor currents were elicited through clamping the cell voltage at -100 mV and perfusing 2 mM GABA onto the tissue sheet for 1–2 seconds. This process was repeated after 15 minutes of 0.1–1.5 μM Alfaxalone perfusion. Percent change responses were expressed relative to the response in the absence of Alfaxalone using the equation [(A-C)/C]*100%, where A represents GABA_A_ receptor current property measurements following acute Alfaxalone treatment and C represents GABA_A_ receptor current property measurements under control conditions [28]. Each point represents data from a separate experiment (*n = 3–7*).
(TIFF)

S1 Dataset Dataset acquired in the analyses for Figs 2, 4, 5, S1 and S2. (XLSX)
