# Supplementary material for: Alfaxalone is an effective anesthetic for the electrophysiological study of anoxia-tolerance mechanisms in western painted turtle pyramidal neurons
Source: PLoS One. 2024 Apr 16;19(4):e0298065. doi: 10.1371/journal.pone.0298065 (PMC11020846; doi:10.1371/journal.pone.0298065)
Supplement: S1 File — (PDF) [file pone.0298065.s001.pdf]

Alfaxalone is an Effective Anesthetic for the Electrophysiological Study of Anoxia-Tolerance Mechanisms in Western Painted Turtle Pyramidal Neurons

Haushe Suganthan[1], Domenic Di Stefano[1], Leslie Buck[1,2]

[1]Department of Cell and Systems Biology, University of Toronto, Toronto, ON, Canada. M5S 3G5;
[2]Department of Ecology and Evolutionary Biology, University of Toronto, Toronto, ON, Canada M5S 3G5

Haushe Suganthan

ABSTRACT

Many anesthetics have long-term effects on ion channels and are not appropriate for same day experimentation. Using whole-cell electrophysiological techniques, we examine the effects of the anesthetic, Alfaxalone, on pyramidal cell action potential amplitude, threshold, rise and decay time, width, frequency, whole cell conductance, and evoked $GABA_A$ receptors currents to determine if any of these characteristics are altered with the use of Alfaxalone for animal sedation.

ATTACHMENTS

Protocol.docx

Protocol Info: Haushe Suganthan, Domenic Di Stefano, Leslie Buck . Alfaxalone is an Effective Anesthetic for the Electrophysiological Study of Anoxia-Tolerance Mechanisms in Western Painted Turtle Pyramidal Neurons . **protocols.io** https://protocols.io/view/alfaxalone-is-an-effective-anesthetic-for-the-elec-c47wyzpe

Created: Nov 15, 2023

Last Modified: Nov 16, 2023

PROTOCOL integer ID: 91094

Funders Acknowledgement:
Leslie Buck
Grant ID: 478124

Animal Care and Cortical Sheet Preparation

1

Note

This study was approved by the University of Toronto Animal Care Committee and conforms to the care and handling of animals as outlined in the Canadian Council on Animal Care's Guide to the Care and Use of Experimental Animals, Vol. 2. Animal use protocol number (20012745). Informed Consent Statement: Not applicable.

Obtain the adult turtles (Chrysemys picta bellii) from Niles Biological and are then housed in a large aquarium equipped with a freshwater recirculating filter system set at 20 °C, a non-aquatic basking platform, and a heat lamp.

2 Maintain the turtles on a photoperiod of 12 hours of light:12 hours of dark and given access to food three times a week.

3 Give the turtles an intramuscular injection of Alfaxalone 10 undetermined stock in hydroxypropyl-beta-cyclodextrin [HP-β-CD] and dissolve in a multi-dose preservative solution (Alfaxan®) (Jurox Pty Ltd; Rutherford, Australia) at 20 undetermined .

4 Once deep anesthesia was achieved, the legs were flaccid, and the eyes were unresponsive to gentle touch, decapitate the animals with a guillotine, and excise the whole brain from the cranium in about 1 minute.

5 Dissect the entire dorsal cortex free and bathed in 3 °C – 5 °C artificial turtle cerebrospinal fluid (aCSF) composed of (in mmol L −1): 97 NaCl, 2.6 KCl, 1.2 $CaCl_2$, 1.0 $MgCl_2$, 2.0 NaH_2PO_4, 26.5 $NaHCO_3$, 20.0 glucose, 5.0 imidazole (adjusted to pH 7.4, and osmolarity 290–300 mOsM).

6 Cut two cortical sheets medially from the visual cortex of each cerebral hemisphere and subdivide into a total of six cortical sheets.

7 Lift the sheets out of the chamber and store them in vials of aCSF for no longer than 48 hrs.

Whole-Cell Electrophysiology Techniques

8 Place the turtle cortical sheets on a cover slip that forms the bottom of an RC-26 open bath perfusion chamber system, with a P1 platform (Harvard Apparatus, Saint-Laurent, QC, Canada).

Note

The chamber was gravity-perfused by a 1-L glass bottle that contained aCSF gassed with 95% O_2/5% CO_2 to achieve oxidative conditions.

9 Conduct the experiments at room temperature (20 °C - 22 °C).

10 Perform the whole-cell recordings of neurons from the dorsal cortex and dorsal medial cortex using fire-polished 5-8 MΩ micropipettes produced from borosilicate glass capillary tubes using a P-97 micropipette puller model (Sutter Instruments, Novato, CA, USA).

Note

The pipette solution contained the following (in mmol L-1): 8 NaCl, 0.0001 $CaCl_2$, 10 Na-HEPES, 110 Kgluconate, 1 $MgCl_2$, 0.3 NaGTP, and 2 NaATP (adjusted to pH 7.4 and osmolarity 290-300 mOsM).

11 Fill the electrode and insert into a 1-HL-U electrode holder attached to a CV-4 headstage (gain: 1/100 U, Axon Instruments, Sunnyvale, CA, USA).

12 Obtain the cell-attached 1-20 GΩ seals using the blind-patch technique.

Note

To achieve a GΩ seal, the recording electrode was advanced towards the cell using a PCS-6000 motorized manipulator (Burleigh, Newton, NJ, USA) until the square-wave pulse abruptly decreased, at which point a slight negative pressure was applied to form a seal.

13 To break into the cell, apply a soft pulse of negative pressure to break through the cell membrane, while the holding potential was voltage-clamped to -70 mV.

14 Once the whole-cell configuration was established, give the cells at least 2 minutes to acclimate to experimental conditions before access resistance was measured, which normally ranged from 20-30 MΩ.

15 Discard the patches if access resistance varied by >25% over the course of an experiment.

16 Collect the data at 5-10 kHz using a MultiClamp 700B digital amplifier, a CV-7B head stage, and a Digidata 1550B interface (Molecular Devices, Sunnyvale, CA, USA), and store on a computer using Clampex 10 software (Molecular Devices, Sunnyvale, CA, USA).

Note

A liquid junction potential (LJP) was accounted for, and experimentally measured between the aCSF and the pipette solution, supported by LJP calculations using a generalized version of the Henderson equation (Clampex junction potential calculator; Molecular Devices, Sunnyvale, CA, USA).

Electrophysiological Identification and Measurement of Actio…

17

Note

Pyramidal neurons were studied and characterized based on electrophysiological properties. In current clamp mode, when current was injected, pyramidal neurons exhibited spike frequency adaptation in response to a sustained current, which was not seen in stellate neurons.

Determine the action potential threshold (APth) by current-clamping cells and injecting current in 10 pA increments in a stepwise manner from sub-threshold for 500 ms until a spike was elicited.

18 Record the threshold at the point at which a sharp elevation in voltage was observed.

19 Measure the full spike amplitude from the point of the APth to the spike tip, while the half-amplitude spike width was measured as the time elapsed between the two-points of the half-amplitude on the spike.

20 Calculate the rise time as the time elapsed between 10% of the full spike amplitude and 90% of the full spike amplitude, while the decay time was calculated as the time elapsed between 10% of the full spike amplitude and 90% of the full spike amplitude.

21 Measure the whole-cell conductance (Gw) as the slope of a voltage-ramp from -120 to -60 mV for 150 ms.

Note

Data measurements for all parameters were made with Clampfit software (Molecular Devices, Sunnyvale, CA, USA).

Impact of acute Alfaxalone application on evoked naïve tissue...

22 To initiate a GABAA receptor current neurons were voltage-clamped at a holding potential of -100 mV, and 2 mM of GABA were applied for 1-2 seconds. These changes resulted in large outward GABAA-receptor currents that were easily detected and differentiated from other currents.

23 From the GABAA receptor currents produced, measure the peak amplitude, baseline holding current, decay time as the 90%-to-10% decay time, and the area under the curve as the integrated area between the measured current and the baseline using Clampfit software (Molecular Devices, Sunnyvale, CA, USA).

24 Perfuse the sheets with oxygenated aCSF and 1 µM Alfaxalone for 15 min, and the GABAA receptor current decay time, integrated area under the curve, peak amplitude, and baseline holding current were measured again.

25 Repeat this procedure utilizing gabazine (25 µM), a GABAA receptor antagonist, rather than Alfaxalone, to see if the currents could be blocked to confirm we were recording GABAA receptor currents. Additionally, Alfaxalone-free stock Alfaxalone solution (vehicle, a generous gift from Jurox; Rutherford, NSW, Australia) had no significant impact when applied alone (n = 4).

Alfaxalone Dose-Response

26 To construct a dose-response curve, GABAA receptor current measurements following acute Alfaxalone treatment were normalized to pre-treatment values to determine the relative change in decay time, area under the curve, and peak amplitude.

27 The same process to determine relative changes in decay time, area under the curve, and peak ampli 15m was repeated for tissue sheets perfused with oxygenated aCSF and [M] 0.1 micromolar (µM) , [M] 0.5 micromolar (µM) , [M] 1 micromolar (µM) or [M] 1.5 micromolar (µM) Alfaxalone for 00:15:00 .

28

Note

GABAA receptor currents were measured as above; however, the recording pipette [Cl-] was increased to 110 mM [Cl-] by equimolar substitution of KCl for Kgluconate, the neurons were voltage-clamped at a holding potential of -100 mV and 2 mM GABA was applied for 15 seconds [26]. This change still resulted in large outward GABAA currents that were detected and differentiated from other currents.

Determine the GABAA receptor current decay time, integrated area under the curve, and peak amplitude as described above.

29 Normalize the decay time, integrated area under the curve, peak amplitude, and baseline holding current to the whole-cell capacitance.

30 Repeat the protocol with cerebral cortex sheets obtained from Alfaxalone-sedated painted turtles at 1 hour, 2 hours, 3 hours, and 5 hours following Alfaxalone administration.

31 Bin the timepoints, so that any measurement performed between 1 and 2 hours was considered 1 hour following whole-animal Alfaxalone exposure, any measurement performed between 2 and 3 hours following whole-animal Alfaxalone exposure was considered 2 hours following whole-animal Alfaxalone exposure, any measurement performed between 3 and 4 hours was considered 3 hours following whole-animal Alfaxalone exposure, and any measurement performed between 5 and 6 hours was considered 5 hours following whole-animal Alfaxalone exposure.

32 Make the measurements on the 30-minute mark were placed in the latter time group.

33 Replace the aCSF solution bathing the Alfaxalone-treated sheets every 30 minutes following the completion of the dissection with the first washout occurring 0.5 hours after Alfaxalone application.
