# Supplementary figures and images for: Alfaxalone is an effective anesthetic for the electrophysiological study of anoxia-tolerance mechanisms in western painted turtle pyramidal neurons
Source: PLoS One. 2024 Apr 16;19(4):e0298065. doi: 10.1371/journal.pone.0298065 (PMC11020846; doi:10.1371/journal.pone.0298065)

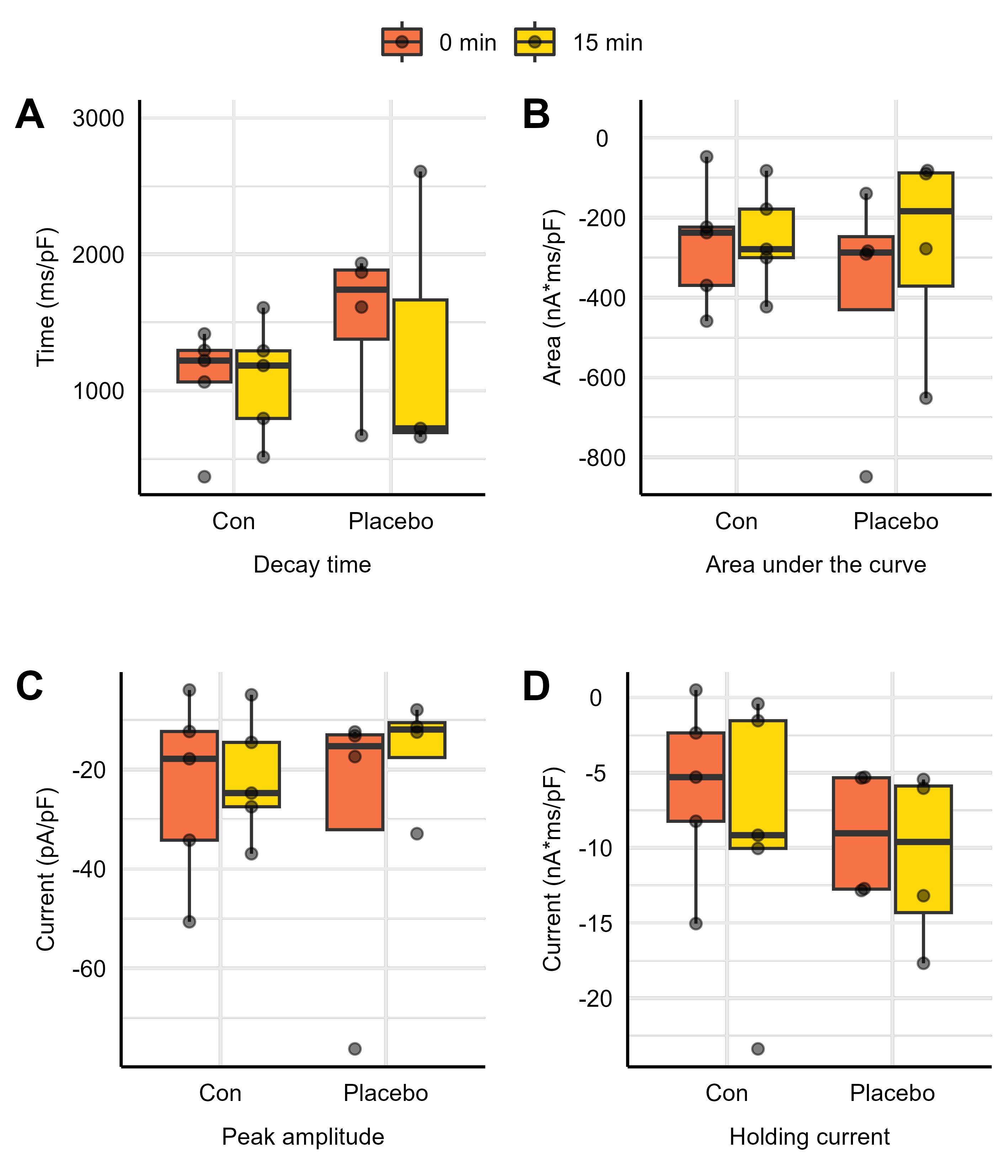

Supplement: S1 Fig — Change in whole cell GABAA receptor response following vehicle application onto tissue. Absolute change in (A) decay time, (B) area under the curve, (C) peak amplitude, and (D) holding current following 15 minutes of vehicle perfusion onto tissue. Each point represents data from a separate experiment (n = 4). (TIFF) [file pone.0298065.s002.tiff]

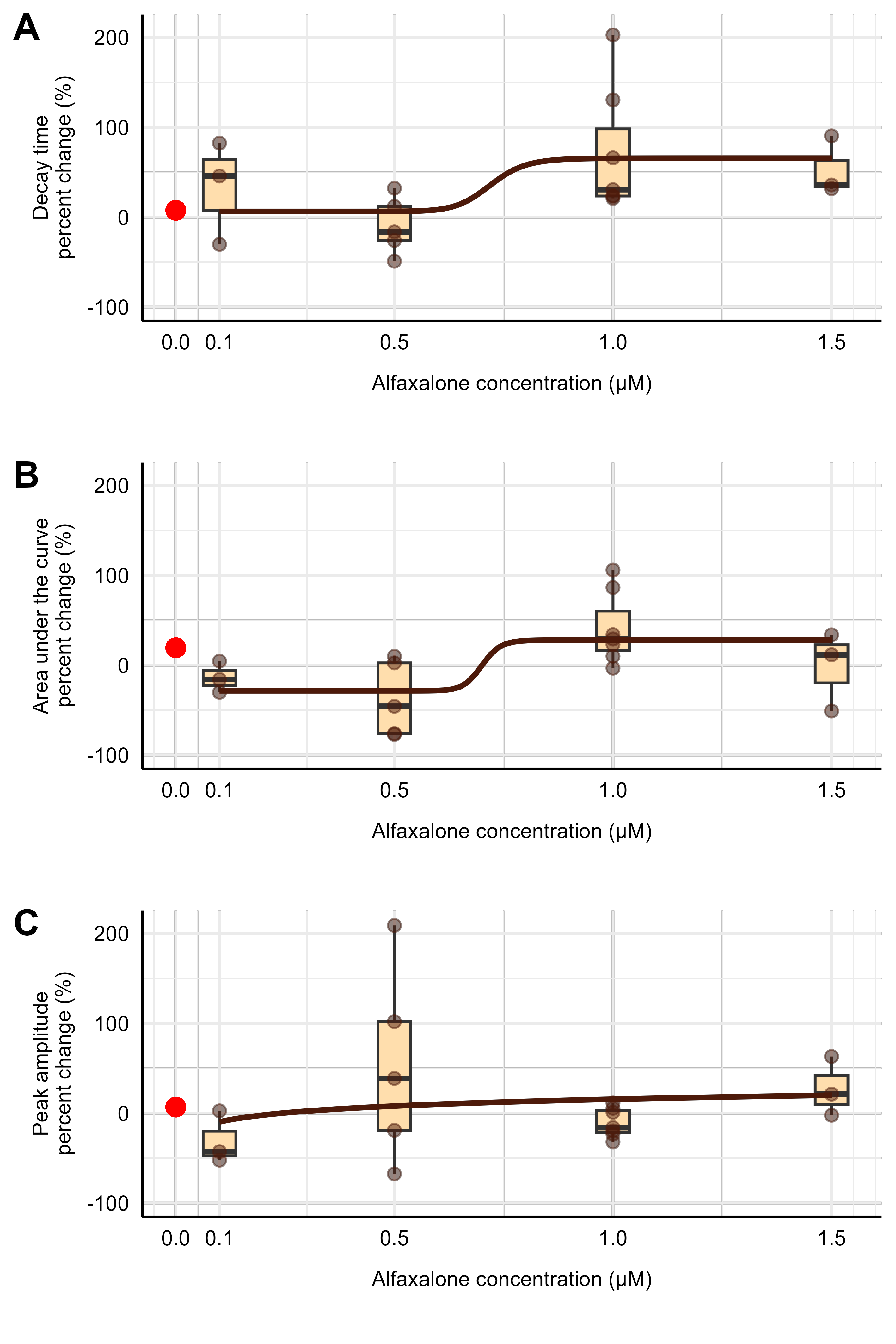

Supplement: S2 Fig — GABAA receptor current measurements following acute Alfaxalone treatment normalized to pre-treatment values to determine the relative change in GABAA receptor current (A) decay time, (B) area under the curve. (C) peak amplitude in naïve tissue sheets perfused with oxygenated aCSF and 0.1 μM (n = 3), 0.5 μM (n = 5), 1 μM (n = 7) and 1.5 μM (n = 3) Alfaxalone for 15 minutes, normalized to control GABAA receptor decay time using the four-parameter Hill equation. The red point represents the average percent change in each property value following 15 minutes of control aCSF perfusion. Minimal peak amplitude potentiation occurred at increasing concentrations of Alfaxalone, resulting in a poor fit to the Hill equation. Alfaxalone concentrations greater than 1.5 μM resulted in patch destabilization within 10 minutes of perfusion. The Alfaxalone-induced potentiation of all properties saturated at approximately 1 μM with minimal patch destabilization. Therefore, it was determined that the acute application of 1 μM Alfaxalone was optimal and would be perfused onto naïve tissue for the remainder of the study. GABAA receptor currents were elicited through clamping the cell voltage at -100 mV and perfusing 2 mM GABA onto the tissue sheet for 1–2 seconds. This process was repeated after 15 minutes of 0.1–1.5 μM Alfaxalone perfusion. Percent change responses were expressed relative to the response in the absence of Alfaxalone using the equation [(A-C)/C]*100%, where A represents GABAA receptor current property measurements following acute Alfaxalone treatment and C represents GABAA receptor current property measurements under control conditions [28]. Each point represents data from a separate experiment (n = 3–7). (TIFF) [file pone.0298065.s003.tiff]
